# Targeting RNA *N*^6^-methyladenosine modification: a precise weapon in overcoming tumor immune escape

**DOI:** 10.1186/s12943-022-01652-3

**Published:** 2022-09-07

**Authors:** Wei Li, Yi Hao, Xingda Zhang, Shouping Xu, Da Pang

**Affiliations:** 1 Harbin Medical University Cancer Hospital, 150 Haping Road, Harbin, 150081 Heilongjiang China; 2 Heilongjiang Academy of Medical Sciences, 157 Baojian Road, Harbin, 150086 Heilongjiang China

**Keywords:** N^6^-methyladenosine (m^6^A), cancer, Tumor, Immunotherapy, Tumor immune escape (TIE)

## Abstract

Immunotherapy, especially immune checkpoint inhibitors (ICIs), has revolutionized the treatment of many types of cancer, particularly advanced-stage cancers. Nevertheless, although a subset of patients experiences dramatic and long-term disease regression in response to ICIs, most patients do not benefit from these treatments. Some may even experience cancer progression. Immune escape by tumor cells may be a key reason for this low response rate. *N*^6^-methyladenosine (m^6^A) is the most common type of RNA methylation and has been recognized as a critical regulator of tumors and the immune system. Therefore, m^6^A modification and related regulators are promising targets for improving the efficacy of tumor immunotherapy. However, the association between m^6^A modification and tumor immune escape (TIE) has not been comprehensively summarized. Therefore, this review summarizes the existing knowledge regarding m^6^A modifications involved in TIE and their potential mechanisms of action. Moreover, we provide an overview of currently available agents targeting m^6^A regulators that have been tested for their elevated effects on TIE. This review establishes the association between m^6^A modifications and TIE and provides new insights and strategies for maximizing the efficacy of immunotherapy by specifically targeting m^6^A modifications involved in TIE.

## Introduction

According to the concept of cancer immunoediting, the immune system has dual roles in preventing and shaping tumors. In immunocompetent hosts, tumor cells obtain three fates: elimination, equilibrium, and escape. Tumor immune escape (TIE) is a process in which the immunologic microenvironment of sculpted tumors can expand in an uncontrolled pathway [1]. Antitumor immunity is mainly mediated by CD8^+^ T cells that specifically recognize antigenic peptides presented by the major histocompatibility complex (MHC, in vertebrates) or class I human leukocyte antigens (HLA-I, in humans) and are activated to kill tumor cells [2]. However, tumor cells can produce inhibitory signals that suppress T-cell function. Immune checkpoint inhibition (ICI) therapy can effectively suppress these signals and reactivate T cells in only a minority of patients. Its limited effectiveness has been demonstrated to be due to reduced antigen presentation, increased immunosuppressive factors, and abnormally activated signaling pathways.

**Fig. 1:**
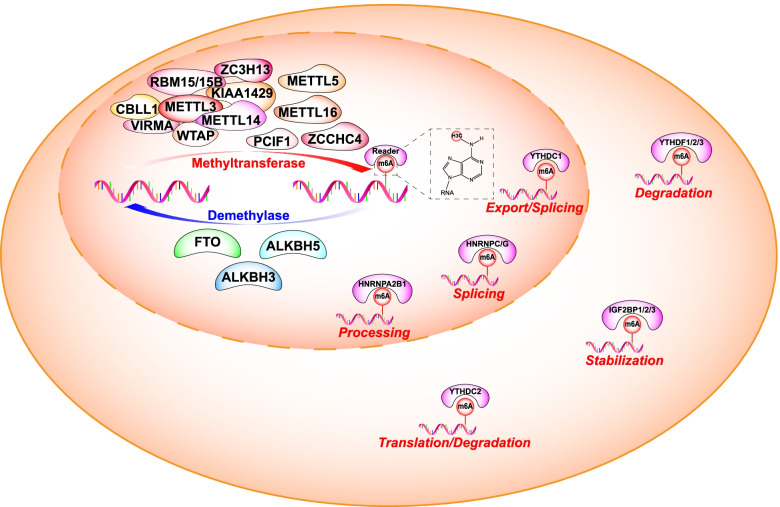
The molecular mechanism of *N*^6^-methyladenosine (m6A) modification. m^6^A modification is a dynamic and reversible epigenetic modification that is regulated by “writers” and “erasers.” It is primarily catalyzed by the m^6^A methyltransferase complex comprising the main components METTL3/METTL14/WTAP and other regulatory proteins (RBM15/15B, KIAA1429, ZC3H13, CBLL1, and VIRMA). In addition, METTL16, METTL5, ZCCHC4, and PCIF1 are methyltransferases that directly catalyze m^6^A modifications in RNA molecules. The erasers mainly consist of FTO, ALKBH5, and ALKBH3. The “readers” are binding proteins that recognize m^6^A modifications in the RNA. m^6^A modification can affect alternative splicing of pre-mRNA, mRNA degradation, mRNA stabilization, miRNA processing, and translation

*N* ^*6*^-methyladenosine (m^6^A) is the most prevalent RNA modification in mammalian cells and was first discovered in 1974 [3]. m^6^A modification sites are evolutionally conserved within a consensus motif DRACH (D = A, G or U; H = A, C, or U), in which A is converted to m^6^A and generally occurs in the coding sequence, 3′ untranslated region (3′ UTR) proximal to the stop codon, and 5′ untranslated region (5′ UTR) of mRNAs [4–6]. It is a potentially reversible and dynamic post-transcriptional modification of RNA molecules that is regulated by methyltransferases (writers) and demethylases (erasers) and recognized by specific binding proteins (readers). The m^6^A modification has significant functions in regulating alternative splicing of pre-mRNAs [5], mRNA degradation [7], mRNA stabilization [8], miRNA processing [9], and cap-independent translation [10] (Fig. 1).

Currently, m^6^A modification involved in TIE is emerging and is expected to be a potential target for improving the limited antitumor immunotherapy response rates. This lack of responsiveness suggests the need to expand the knowledge of m^6^A modifications in TIE regulation. Furthermore, an in-depth understanding of the underlying mechanism of action will contribute to developing new combination therapies to promote antitumor immunotherapy. This review describes the existing evidence regarding m^6^A modifications, their influence on TIE, and their underlying molecular regulatory mechanisms. Moreover, we discuss available agents targeting m^6^A modifications to improve the efficacy of immunotherapy. We hope this review will broaden our understanding of the association between m^6^A modifications and TIE and provide new insights into therapeutic strategies targeting m^6^A involvement in TIE.

### *N*^6^-methyladenosine (m^6^A) regulators

#### Writers

The deposition of m^6^A is mainly catalyzed by a methyltransferase complex (MTC) comprising numerous components. As a core component of MTC, methyltransferase-like protein 3 (METTL3) is an S-adenosyl methionine (SAM)-binding protein that catalyzes the transfer of methyl groups in SAM to adenine bases in RNA, METTL14 stabilizes the structure of MTC and identifies the consensus motif DRACH, and Wilms tumor 1-associated protein (WTAP) promotes the recruitment of METTL3 and METTL14 [11, 12]. In addition to the MTC, several other enzymes act as independent methyltransferases: METTL16 catalyzes m^6^A on U6 snRNA, ncRNAs, and pre-RNAs [13], zinc finger CCHC-type containing 4 (ZCCHC4) deposits m^6^A on 28S rRNA [14], METTL5 adds m^6^A to 18S rRNA [15, 16] and phosphorylated CTD interacting factor 1 (PCIF1) catalyzes both m^6^A and a different RNA modification: *N*^6^,2′-O-dimethyladenosine (m^6^Am) [17–19]. However, with the advent of m^6^A-Crosslinking-Exonuclease-sequencing (m^6^ACE-seq), a newly developed technique for quantitative single-base-resolution sequencing of m^6^A and m^6^Am, which quantitatively map precise locations of transcriptome-wide m^6^A/m^6^Am in cells [20]. This technique can compensate for the shortcomings of past techniques, such as the poor resolution (~ 150 nt) of methylated RNA immunoprecipitation sequencing (MeRIP-seq), the time-consuming and inconvenient procedures (such as radioactive gel electrophoresis) of m^6^A individual-nucleotide-resolution cross-linking and immunoprecipitation (miCLIP), and their inability to detect m^6^A between different samples. It is argued that METTL16 does not methylate m^6^A directly at its specific ‘UACm^6^AGAGAA’ motif but controls intracellular SAM levels [20]. Knockdown of *METTL16* resulted in the loss of m^6^A in the transcriptome, except for METTL16 directly methylated sites. Motif analysis also indicated that sequences located at the METTL16-dependent m^6^A sites delineate the METTL3-dependent “DRm^6^ACH” motif. *METTL16* knockdown also reduced m^6^A at all 5 ‘UACAGAGAA’ sites and ‘UACAGAAAA’ sites within the 3′ UTR of *Mat2a* transcript, which encodes a SAM synthetase. All these results suggest that METTL16 may catalyze m^6^A through an indirect mechanism, likely via regulating MAT2A expression to control intracellular SAM levels.

#### Erasers

Fat mass and obesity-associated protein (FTO) is the first demethylase discovered in 2011, and another demethylase, Alkb homolog 5 (ALKBH5), was discovered 2 years later [21, 22]. Recent studies in 2017 have identified ALKBH3 as a demethylase responsible for removing m^6^A on transfer RNA (tRNA) [23]. All three demethylases belong to the alpha-ketoglutarate-dependent dioxygenase family and catalyze m^6^A demethylation in an Fe (II)- and α-ketoglutaric acid-dependent manner. With the discovery of these demethylases, m^6^A is considered a dynamic reversible process; however, questioned in further studies. FTO is primarily and potentially exclusively located in the nucleus [21, 24]. Therefore, FTO can demethylate m^6^A only within a short window after biogenesis in the nucleus, while it cannot regulate m^6^A of cytosolic mRNAs. Thus, FTO-mediated demethylation cannot be dynamic for cytoplasmic mRNAs. Furthermore, individual depletion of ALKBH5 and using m^6^ACE-seq to estimate m^6^A accumulation showed increased m^6^A accumulation in the DRACH motifs. They found that ALKBH5 maintains the DRACH sites constantly and completely unmethylated rather than reverse methylation. Thus, FTO keeps m^6^Am sites unmethylated and suppresses m^6^A of mRNA before it shuttles out of the nucleus [20]. This indicated that m^6^A is not reversible in the presence of demethylases. More importantly, several studies have argued that the physiologic target of FTO is not m^6^A but m^6^Am [25]. First, several studies found that once the m^6^A is deposited, it cannot be removed [26–28]. Second, the reaction rates of FTO toward m^6^A are low [21]. Third, the m^6^A demethylation function of FTO is not sequence-specific [29–33]. Lastly, transcriptome-wide analysis of *FTO* knockdown does not show a robust increase in m^6^A [34]. A detailed analysis of m^6^A peak intensities in wild-type and *FTO* knockdown mice to investigate the reasons for the lack of the global increase in m^6^A levels when *FTO* was knocked down. The results showed that the m^6^A peak exhibits a 5′ UTR increase which is the site of m^6^Am. Further study also proved that FTO had nearly 100 times higher catalytic activity against m^6^Am compared to m^6^A, suggesting m^6^Am as the indeed substrate [25, 35]. Furthermore, FTO is demonstrated to demethylate m^6^Am of snRNA which may influence mRNA splicing [36]. This finding is confirmed by an antibody-independent technique MAZTER-seq, that neither knockdown nor overexpression of *FTO* impact the m^6^A levels in human embryonic stem cells and HEK293T cells. However, overexpressed ALKBH5 induces a subtle decrease in methylation levels [37]. However, many studies have demonstrated that FTO acts on specific m^6^A sites and impacts targeted genes in cancer. This discrepancy may be due to the limitations of the detection and statistical methods used in previous studies, which could not distinguish between m^6^A and m^6^Am. In contrast, demethylases may be recruited to specific transcripts or induced cytosolic translocation to activate the demethylation program in some cancers [38, 39]. However, FTO does not affect global m^6^A, possibly because it affects certain gene expressions that promote m^6^A. Overall, m^6^A demethylation is rare, and the reversibility is unlikely to be a major mechanism in most cells.

#### Readers

The m^6^A recognized by different readers can execute diverse downstream biological functions (Fig. 1). m^6^A is mainly recognized by the YT521-B homology domain-containing proteins (including YTHDC1, YTHDC2, YTHDF1, YTHDF2, and YTHDF3), which has an aromatic cage to specifically accommodate methyl mark [40, 41]. YTHDC1 is broadly expressed and is predominantly localized in the nucleus, which regulates RNA export and pre-mRNA splicing [42, 43]. In contrast, the YTHDC2 expression is tissue-restricted and located in both nuclear and cytosolic, regulating mRNA stabilization and translation [7]. YTHDC2 also has an N-terminal R3H domain, a C-terminal YTH domain, and a helicase core module with sequence motifs characteristic of superfamily 2 DExHbox helicases, an ankyrin repeat pair insertion, and two C-terminal HA2 and OB domain extensions, which confers YTHDC2 helicase activity. Studies have shown that YTHDC2 plays a critical role in the meiotic entry process by interacting with m^6^A-modified transcripts and reducing their stability or enhancing translation [44–47]. However, Jain et al. argued that the helicase activity of YTHDC2 plays a role in spermatogenesis rather than recognizing m^6^A. To lay down the principles underlying its molecular function, they found that mutation of the m^6^A binding pocket of YTHDC2 has no significant effect on gametogenesis and mouse fertility. Cross-linking and immunoprecipitation (CLIP) data suggested that YTHDC2 binds to the site containing U-rich and UG-rich motifs and affects their steady-state levels rather than binds to m^6^A-modified sites to regulate translation. Mutation of the ATPase motif in the helicase domain of YTHDC2 did not affect meiotic entry. However, it blocked the progression of meiotic prophase I, causing catastrophic failure of spermatogenesis and leading to sterility [48]. It may be consistent that YTHDC2 recruits 5′-3′ RNA exonuclease XRN1 as a partner to reduce the target RNA stability [47]. This provides insight into how gene expression regulation has diversified over the course of evolution.

The YTHDF family contains three paralogs, YTHDF1, YTHDF2, and YTHDF3, each of which has been reported to have different functions with limited redundancy. YTHDF1 promotes mRNA translation, YTHDF2 enhances mRNA degradation, and YTHDF3 cooperates with YTHDF1 and YTHDF2 to accelerate translation and degradation [49–52]. However, the latest research proved that the YTHDF proteins enhance mRNA degradation in a redundant manner rather than translation efficiency [53]. Zaccara et al. proposed a distinctly unified model that all m^6^A sites bind all three YTHDF proteins to promote mRNA degradation in an m^6^A-dependent manner in Hela cell and leukemia cell line models rather than enhance translation. This redundancy may be due to the similar characteristics of YTHDF proteins, including RNA-binding properties, m^6^A binding preferences and affinities in transcriptome, high confidence with RNA degradation machinery, and intracellular localization. The mRNA degradation efficiency was highest when all three proteins were present, and compensation cannot occur when all these proteins are depleted; the mRNA is the most stable. Notably, the previous studies that YTHDF paralogs promote translation, which was affected by bioinformatic and technical issues, may be incorrect [53]. Together, these diverse lines of evidence suggest that YTHDF proteins do not promote translation; however, we cannot exclude their role in promoting translation in other cell lines or conditions. Because YTHDF proteins show significantly different expression levels in different tissues, their ability may also be affected by different phosphorylation modes, specific stimuli, and cellular contexts [49, 54–58]. Lasman et al. found that this functional compensation is context-dependent, which affects mRNA stability via an m^6^A-dependent manner rather than translation [59]. They explored the effects of YTHDF proteins in vivo in mouse gametogenesis, postnatal viability, and in vitro in mouse embryonic stem cells (mESCs). Only YTHDF2 is crucial for mouse gametogenesis, which may be due to the different expression spatial or temporal space, cell types, and intracellular localization. YTHDF proteins show compensation for one another in a dosage-dependent manner during embryonic development and gestation. In mESCs, only triple knockout of YTHDF paralogs shows the effect on mRNA decay and differentiation.

In addition to these canonical readers, other proteins also act as indirect m^6^A readers with a distinct binding mechanism, including insulin-like growth factor 2 mRNA binding proteins (IGF2BPs) (including IGF2BP1, IGF2BP2, and IGF2BP3) and RNA binding proteins (RBPs) (including HNRNPA2B1, HNRNPC, and HNRNPG) [60–62]. Instead of directly recognizing and binding to m^6^A, these proteins bind to target RNAs via an indirect mechanism termed “m^6^A-switch”, that m^6^A can alter its local RNA structure and enhance the accessibility of its base-paired residues or nearby regions to modulate protein binding. The m^6^A-switch is proven to regulate HNRNPC binding activities, affecting target mRNAs’ abundance and alternative splicing, demonstrating the regulatory role of m^6^A-switch on gene expression and RNA maturation [60]. The HNRNPA2B1 protein was initially proposed as a direct m^6^A mark reader interacting with the consensus motif via its RNA-recognition motifs (RRMs) and regulating alternative splicing [63]. However, Wu et al. found that HNRNPA2B1 may mediate the effects of m^6^A via the m^6^A-switch mechanism [62]. Their structural study showed no aromatic cage-like surface in crystal structures. HNRNPA2B1 does not recognize or show enhanced binding to the m^6^A-modified RNA substrates in vitro. In vivo study showed that only a small fraction of the m^6^A nuclear sites is located near HNRNPA2B1. Therefore, the m^6^A-switches may account for the enhanced HNRNPA2B1 binding to m^6^A [61]. Moreover, HNRNPG is also proved an indirect reader to use the m^6^A-switch mechanism, which binds to m^6^A-modified RNAs by a low-complexity region [62].

The three IGF2BP paralogs are highly similar, with two N-terminal RRMs and four C-terminal K-Homology (KH) domains, characterized by a conserved αβ-topology that can be structurally and functionally included in two di-domains (KH1-2 and KH3-4) [64]. Functionally, IGF2BP1-3 fortifies the stability and increases m^6^A-modified mRNAs translation efficiency [8]. Nevertheless, the latest research takes a different view of their roles as m^6^A readers. Sun et al. used the in vivo click selective 2-hydroxyl acylation and profiling experiments (icSHAPE), a technique they developed to map RNA structure in three compartments – chromatin, nucleoplasm, and cytoplasm, to help determine the precise relationship between RNA structure and cellular processes, including transcription, translation, RNA decay, RBP interaction, and RNA modification. They used the cytotopic RNA structurome data to filter published CLIP-seq data and calculated m^6^A modification effect on protein binding. The results showed that while the canonical readers bind most strongly to the m^6^A sites, HNRNPC and IGF2BP protein binding peaks at a distance. RNA pull-down experiment showed that IGF2BP3 exhibits enhanced binding to the m^6^A-modified RNAs and uracil mutations compared to the unmethylated RNAs. This suggests that IGF2BP proteins may also be able to read the structural changes induced by m^6^A-switch [65].

### Impaired antigen processing presentation and tumor immune escape (TIE)

Tumor antigens (tAgs) are expressed on the surface of cells by HLA-I and undergo two distinct pathways to induce an effective antitumor response. First, tAgs are taken up by dendritic cells (DCs) and cross-presented to prime naive CD8^+^ T cells [66]. Second, tAgs are directly presented by tumor cells to be recognized and killed by primed CD8^+^ T cells. Under constant T-cell selection, tumor cells can avoid immune recognition via different evasion mechanisms [67]. In both pathways, tumors impair antigen presentation by disrupting DC function or reducing HLA-I expression to escape immune recognition, eventually leading to TIE.

#### Dendritic cell defects and TIE

**Fig. 2:**
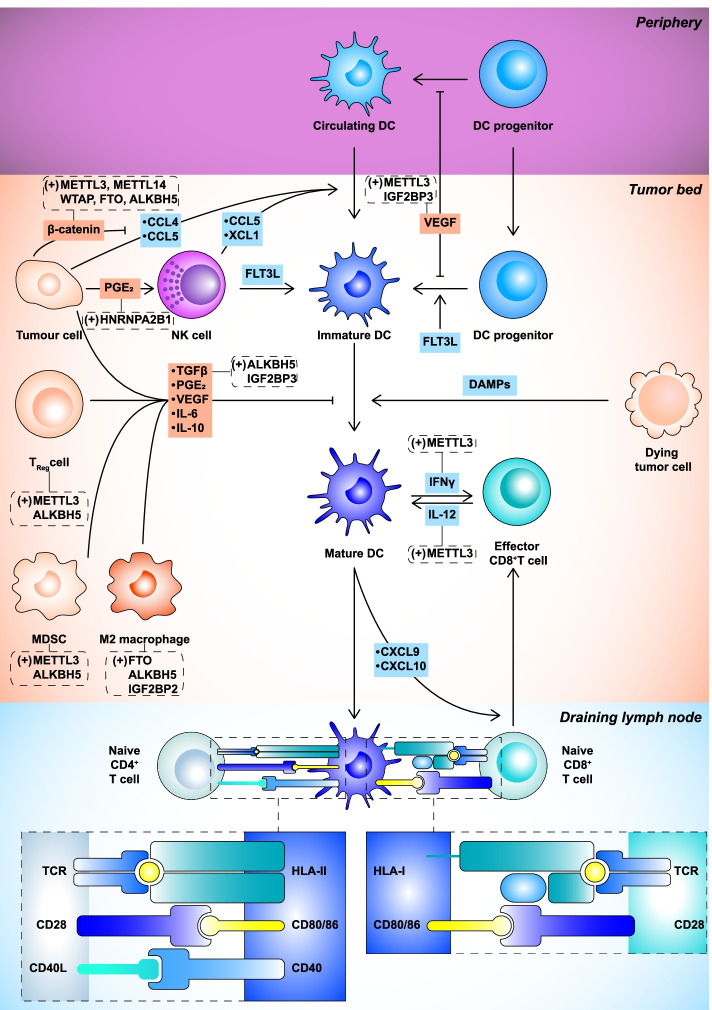
Dendritic cells (DCs) in antitumor immunity. DCs are recruited into the tumor bed by chemokines, such as CC chemokine ligands 4 (CCL4), CCL5, and XC-chemokine ligand 1 (XCL1). FMS-like tyrosine kinase 3 ligand (FLT3L) promotes the differentiation and survival of DCs. Immature DCs take up dying tumor cells that release damage-associated molecular patterns, migrate to the draining lymph nodes, and process and load cancer antigens onto human leukocyte antigen (HLA)-I and HLA-II for presentation to CD8^+^ and CD4^+^ T cells, respectively. Naive CD4^+^ T cells are primed first, which allows DCs to prime CD8^+^ T cells via CD40-CD40L signaling. Moreover, intratumoral DCs generate chemokines CXC-chemokine ligand 9 (CXCL9) and CXCL10 to recruit effector CD8^+^ T cells from draining lymph nodes. Tumors can change DC functions to achieve tumor immune escape. Vascular endothelial growth factor (VEGF) prevents DC differentiation and maturation. Activation of β-catenin signaling and expression of prostaglandin E_2_ (PGE_2_) prevent the recruitment of DCs to the tumor bed by blocking chemokine secretion, including CCL4, CCL5, and XCL1. PGE_2_ prevents the recruitment and maturation of DCs. Tumor cells, CD4^+^ regulatory T cells (Treg), myeloid-derived suppressor cells (MDSCs), and M2 macrophages produce cytokines, including tumor growth factor-β (TGFβ), interleukin (IL)-6, IL-10, PGE_2_, and VEGF, to prevent DC maturation. CCL4, CC-chemokine ligands 4; CXCL9, CXC-chemokine ligand 9; DAMP, damage-associated molecular pattern; DC, dendritic cell; FLT3L, FMS-like tyrosine kinase 3 ligand; HLA-I, class I human leukocyte antigen; IL-6, interleukin-6; MDSC, myeloid-derived suppressor cell; PGE_2_, prostaglandin E_2_; TGFβ, transforming growth factor-β; Treg cell, regulatory T cell; VEGF, vascular endothelial growth factor; XCL1, XC-chemokine ligand 1.

DCs take up dying tumor cells that release dangerous signals, undergo maturation, migrate to the draining lymph nodes, and process and load tAgs onto HLA-I to CD8^+^ T cells (Fig. 2). METTL3-mediated m^6^A modification was demonstrated to promote DC maturation and activation in an innate immune response model. Mechanistically, *CD40*, *CD80*, and TLR4 signaling adaptor *Tirap* transcripts are methylated by METTL3, promoting their translation in DC to enhance T cell activation and cytokine production [68]. In contrast, m^6^A modification generally displays the opposite role in antitumor immunity models. In melanoma and hepatocellular carcinoma (HCC), activating the β-catenin signaling pathway can prevent the infiltration of DCs and CD8^+^ T cells within the tumor microenvironment (TME) by inhibiting the secretion of the CC chemokine ligands CCL4 or CCL5, which directly impairs the therapeutic benefit of ICI [69, 70]. Numerous studies have demonstrated that m^6^A modifications can activate the β-catenin signaling pathway in different cancers (Table 1). For example, m^6^A modification promotes the stability of *circRNA-SORE* mRNA, which enhances sorafenib resistance through the circRNA-SORE/miR-103a-2-5p/miR-660-3p/Wnt2b/β-catenin pathway in HCC [72]. YTHDF2 decreases *lncAY* levels in an m^6^A-dependent manner, which promotes the proliferation and migration of HCC cells via the lncAY/BMI1/Wnt/β-catenin signaling pathway [85]. These studies have demonstrated that m^6^A modification promotes progression and drug resistance by activating the β-catenin signaling pathway in various cancer types. We envision that m^6^A may promote TIE by activating the β-catenin signaling pathway, which needs further studies. Prostaglandin E_2_ (PGE_2_) secretion in a cyclooxygenase 2 (COX2)-dependent manner can impair the accumulation and viability of DCs in tumors and suppress DC maturation [87–89]. In lung cancer (LC), HNRNPA2B1 upregulates COX2 expression, PGE_2_ production, and promotes tumor growth [90]. Moreover, m^6^A modification induces TIE by increasing antigen degradation in DCs [91]. m^6^A modifies transcripts encoding lysosomal proteases to promote lysosomal cathepsins translation in DCs, which is associated with enhanced ingested antigens degradation [92, 93]. In summary, m^6^A modification plays a vital role in regulating the cross-presentation function of DCs and priming of CD8^+^ T cells, including maturation, migration, activity, and antigen degradation of DCs.

**Table 1:** m^6^A modification promote TIE by activating β-catenin signaling pathway

Regulator	Cancer	Mechanism	Functions	Refs.
METTL3	CRC	Stabilizes *Sec62.*	Promotes stemness and chemoresistance of CRC cell by activating β-catenin pathway.	[71]
METTL3, METTL14, FTO, ALKBH5	HCC	Stabilizes *circRNA-SORE*.	Induces sorafenib resistance of HCC by activating β-catenin pathway.	[72]
METTL3	hepatoblastoma	Promotes *CTNNB1* stability.	Promotes proliferation and colony-forming ability of hepatoblastoma cell by activating β-catenin pathway.	[73]
METTL3	hepatoblastoma	Promotes *β-catenin* expression.	Promotes hepatoblastoma cell proliferation, invasion, and migration by activating β-catenin pathway.	[74]
METTL3	melanoma	Promotes *UCK2* stability.	Promotes melanoma cell invasion by activating β-catenin pathway.	[75]
METTL3	NPC	Stabilizes *Tankyrase.*	Promotes NPC cell migration and invasion by activating β-catenin pathway.	[76]
METTL3	EC	Promotes *APC* degradation.	Promotes aerobic glycolysis, proliferation, and tumor formation in mice by activating β-catenin pathway.	[77]
METTL3	osteosarcoma	Promotes *LEF1* stability.	Promotes osteosarcoma cell proliferation, migration, and invasion by activating β-catenin pathway.	[78]
METTL3	NPC	Promotes *TRIM11* stability.	Enhances the chemoresistance by activating β-catenin pathway.	[79]
FTO	CC	Upregulates *β-catenin*.	Promotes chemo-radiotherapy resistance by upregulating β-catenin.	[80]
ALKBH5	glioblastoma	Promotes *SOX2* expression.	Promotes glioblastoma cell proliferation, inhibits apoptosis and temozolomide sensitivity by activating β-catenin pathway.	[81]
YTHDF1	GC	Promotes *FZD7* translation.	Promotes GC cell proliferation and tumorigenesis via β-catenin pathway.	[82]
YTHDF1	CRC	Promotes *TCF7L2/TCF4* translation.	Promotes intestinal stem cell stemness via β-catenin pathway.	[83]
YTHDF1	HCC	Promotes *FZD5* translation.	Promotes HCC cell proliferation and metastasis via β-catenin pathway.	[84]
YTHDF2	HCC	Promotes *lncAY* expression.	Promotes HCC cell proliferation and migration via β-catenin pathway.	[85]
YTHDF2	LC	Promotes *AXIN1* decay.	Promotes LC cell proliferation and metastasis via β-catenin pathway.	[86]

*Abbreviations: CC* cervical cancer, *CRC* colorectal cancer, *EC* esophageal cancer, *GC* gastric cancer, *HCC* hepatocellular carcinoma, *LC* lung cancer, *NPC* nasopharyngeal cancer

#### Defective class I human leukocyte antigen presentation in tumors

**Fig. 3.**
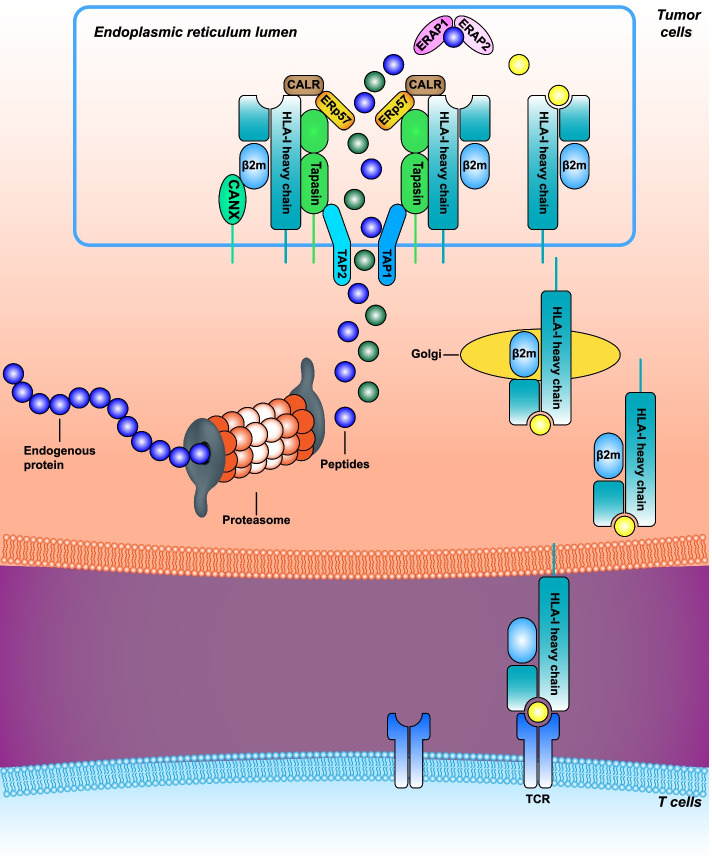
Overview of human leukocyte antigen (HLA)-I antigen processing and presentation machinery. Peptides are generated by the degradation of endogenous proteins via the proteasomal pathway. The peptides are then translocated by transporters associated with the antigen processing (TAP) in the endoplasmic reticulum (ER). In the ER lumen, peptides can be further trimmed by ER aminopeptidase 1 (ERAP1) and ERAP2. The peptide-loading complex, comprising ERp57 and calreticulin (CALR), helps the loading and folding of HLA-I molecules with peptide and β2-microglobulin (β2M). Tapasin assesses peptides for stable binding to the complex formed by the HLA-I heavy chain α1 and α2 domains. CALR, calreticulin; ER, endoplasmic reticulum; ERAP1, ER aminopeptidase 1; HLA-I, human leukocyte antigen-I; TAP, transporters associated with antigen processing; β2M, β2-microglobulin

After priming with DCs, CD8^+^ T cells migrate from the draining lymph nodes to the tumor and recognize the tAgs present on HLA-I by the tumor cells for elimination. In addition to the failure in the cross-presentation function of DCs, malfunctions also occur in the tAg presentation pathway of tumor cells. The antigen processing and presenting machinery (APM) in tumor cells mainly consists of the ubiquitin-protease system and HLA-I complex. The presentation processes are divided into four steps: (i) processing proteins into peptides, (ii) peptide transportation, (iii) installation of peptides on the HLA-I complex, and (iv) peptide-HLA-I complex translocation and presentation (Fig. 3). m^6^A modification has been reported to be correlated with APM in tumors, such as HCC, pancreatic cancer (PC), esophageal cancer (EC), and breast cancer (BC) [94–97]. In BC, higher m^6^A modification levels are associated with elevated expression of HLA-A and more tumor-infiltrating CD8^+^ T cells, helper T cells, and natural killer cells, but decreased expression of programmed cell death protein ligand 1 (PD-L1), PD-L2, T-cell immunoglobulin and mucin-domain containing 3 (TIM3), and CCR4. Lower m^6^A modification is related to the hallmarks of PI3K/AKT signaling in cancer, KRAS signaling, angiogenesis, and shorter overall survival (OS) [97]. In HCC, downregulated METTL3 expression is related to increased MHC, co-stimulatory, and adhesion molecules [94]. However, current studies are based on the analysis of public databases that require further experimental verification.

### Immunosuppressive cells and TIE

#### CD4^+^ regulatory T cells (Tregs) and TIE

Based on different antigen signals and cytokine stimulations, CD4^+^ T cells can differentiate into numerous subtypes, such as Tregs and helper T cells 1, 2, and 17 (Th1, Th2, and Th17). Tregs generally play pro-tumor roles, are immunosuppressive, and are associated with TIE [98]. Tregs inhibit the function of antitumor T cells by producing inhibitory cytokines, including interleukin (IL)-10, IL-35, and TGF-β [99]. Tregs can also inhibit the activation of CD8^+^ T cells by expressing cytotoxic T lymphocyte antigen 4 (CTLA4), an inhibitory molecule [98]. Tregs inhibit T-cell activation by blocking the maturation and function of DCs [99]. Studies have shown that METTL3 regulates T-cell homeostasis and differentiation in mouse T cells. METTL3-meditated m^6^A modification promotes mRNA degradation of SOCS family genes and enhances T-cell differentiation and proliferation via the IL7R/JAK/STAT5 pathway [100]. In a further study, they demonstrated in vivo that METTL3-mediated m^6^A modification promotes the IL2-STAT5 pathway and immunosuppressive functions of Tregs [101].

#### Myeloid-derived suppressor cells (MDSCs) and TIE

MDSCs are potent immunosuppressive cells in cancer, promote tumor initiation and metastasis, and have attracted attention as targeted therapeutic interventions [102]. MDSCs induce TIEs via various pathways. First, MDSCs significantly inhibit MHC II expression, cross-presentation, DC activation, and T-cell stimulation by upregulating indoleamine 2,3-dioxygenase (IDO) and myeloperoxidase. Second, increased IDO levels enhance the differentiation and suppressive functions of Tregs. Moreover, the IDO-induced depletion of tryptophan attenuates cytotoxic T lymphocyte (CTL) proliferation. Furthermore, upregulated arginase 1 causes CTL disability and apoptosis [103]. In CC, the expression of METTL3 and CD33^+^ MDSCs in tumor tissues is higher than that in adjacent normal tissues, and their expression is positively correlated. Their levels in tumor samples are significantly related to poor disease-free survival (DFS) and OS. More importantly, the expression of METTL3 is an independent factor for DFS and OS in patients, whereas the number of CD33^+^ MDSCs is an independent predictor of DFS. Furthermore, knockdown of METTL3 potently reduces CD33^+^ CD11b^+^ HLA-DR^−^ MDSCs and tumor-derived MDSCs in CD33^+^ or HeLa cells [104].

#### Tumor-associated macrophages (TAMs) and TIE

Macrophages are myeloid cells with various phenotypes, of which M1 and M2 subtypes are extreme. M1 can kill tumor cells by emerging as antigen-presenting cells (APCs) or generating nitric oxide, type 1 cytokines, and chemokines. M2 macrophages are activated by IL-4, IL-13, TGF-β, and/or glucocorticoids. M2 macrophages produce cytokines and type II chemokines to accelerate tumor growth. In turn, stromal and tumor-associated factors in the TME can polarize macrophages to the M2 type, particularly the TAM type, to drive TIE. In BC, M2 macrophages produce high levels of IL-10, which effectively attenuate CD8^+^ T cell-dependent responses to paclitaxel by downregulating IL-12 in intratumoral DCs [105]. FTO has been demonstrated to promote both M1 and M2 polarizations. Gu et al. found that *FTO* knockdown reduced the mRNA stability of *STAT1* in M1 and *PPAR-γ* in M2 in a YTHDF2-dependent manner [106]. Furthermore, IGF2BP2 has been reported to shift M1 macrophages to M2 activation by stabilizing *TSC1* and *PPAR-γ* in an m^6^A-dependent manner [107]. ALKBH5 was demonstrated to promote macrophage recruitment, and M2 polarization and phagocytosis in glioblastoma multiforme cells and induce immunosuppression in allograft tumors. ALKBH5-mediated m^6^A demethylation stabilizes *NEAT1*, leading to TAM recruitment and TIE via ALKBH5/NEAT1/CXCL8/IL-8 pathway [108].

In summary, immunosuppressive Tregs, MDSCs, and M2 macrophages can help tumors obtain TIE in different ways, which prevents the immune system from recognizing and destroying the tumor. These immunosuppressive cells are regulated by m^6^A modification to obtain TIE, which may be a promising target for antagonizing TIE and reinforcing the effect of antitumor immunotherapy.

### Immune checkpoint molecules and TIE

Immune checkpoints have emerged as a large number of inhibitory pathways in the immune system that are critical for maintaining self-tolerance and regulating the duration and magnitude of the peripheral tissue physiological immune response to reduce collateral tissue damage. Tumors acquire TIE through these immune checkpoint pathways [109]. Co-inhibitory and co-stimulatory receptor signaling pathways play crucial roles in T cell activation, differentiation, effector function, and survival. Therefore, promising therapeutic approaches to reinvigorate a T-cell response can be achieved using inhibitors of co-inhibitory factors or agonists of co-stimulatory factors. The currently known co-inhibitory receptors include CTLA4, programmed cell death protein 1 (PD-1), PD-L1, lymphocyte activation gene 3, TIM3, and T cell immunoreceptor with immunoglobulin and ITIM domain (TIGIT). In addition, the co-stimulatory factors include B- and T-cell lymphocyte attenuator, glucocorticoid-induced tumor necrosis factor (TNF) receptor family-related protein, OX40, 41BB, and inducible T cell co-stimulatory. Next, we review the research progress on m^6^A in immune checkpoint regulation.

The discovery and clinical implementation of ICIs targeting CTLA4, PD-1, and PD-L1 have revolutionized cancer treatment and have been recognized by the 2018 Nobel Prize for Medicine and Physiology. PD-L1, also known as B7 homolog 1 or cluster of differentiation 274, is the first functionally characterized ligand of co-inhibitory PD-1. PD-L1 is a transmembrane protein expressed in various tissues, but it is primarily found in T cells, B cells, DCs, monocytes, and various tumor cells. However, PD-1 is mainly expressed on the surfaces of activated T cells, B cells, and DCs. T cells are the basis of the immune response, and their activation requires the interaction of two signals: (i) via the CD3 complex upon the binding of the T-cell receptor (TCR) expressed on the surface of T cells with HLA-I and its cognate peptide antigen on APCs or a target cell and (ii) another co-stimulatory receptor, CD28, which binds to the B7 family of co-stimulatory molecules mainly expressed on APCs. T cells differentiate, proliferate, produce cytokines, and subsequently form memory T cells [110]. PD-1 interacts with its ligand PD-L1, leading to the dephosphorylation of downstream TCR signaling molecules and inhibition of TCR-mediated IL2 production and T-cell proliferation, which causes TIE [111]. Currently, anti-PD-1/PD-L1 antibodies are approved for use in patients with a wide range of tumor types [112]. Antibodies against PD-1 include nivolumab, pembrolizumab, and cemiplimab. The PD-L1 antibodies include durvalumab, atezolizumab, and avelumab. m^6^A modification has been found to modulate PL-L1/PD-1 expression and promote immunosuppression, thus contributing to TIE [38, 113–115]. Knockdown of m^6^A regulators or targeted inhibitors can potently enhance the immunotherapeutic effects of these antibodies. ALKBH5 was verified to promote *PD-L1* expression, consequently reshaping TME and affecting immunotherapy efficacy in intrahepatic cholangiocarcinoma. Knockdown of *ALKBH5* increases m^6^A modification in the 3’UTR of *PD-L1* mRNA, thereby promoting its degradation in a YTHDF2-dependent manner. Moreover, ALKBH5 inhibits the expansion and cytotoxicity of T cells by promoting *PD-L1* expression. Single-cell mass cytometry analysis showed that ALKBH5 promotes PD-L1 expression in monocytes/macrophages and reduces the infiltration of MDSCs. Analysis of samples from patients receiving anti-PD1 immunotherapy showed that tumors with strong nuclear expression patterns of ALKBH5 are more sensitive to PD-L1 immunotherapy [115]. FTO promotes melanoma tumorigenesis and inhibits anti-PD-1 blockade immunotherapy. *FTO* knockdown increases m^6^A modification of *PD-1* (*PDCD1*), *CXCR4*, and *SOX10*, contributing to enhanced RNA decay via the m^6^A reader YTHDF2 in melanoma cells. It has been consistently proven that loss of FTO sensitizes melanoma cells to interferon-γ and sensitizes melanoma to anti-PD-1 treatment in mice, which depends on adaptive immunity [38]. In addition to PD-1/PD-L1, other immune checkpoint molecules are also associated with m^6^A modifications and related regulators, such as CTLA-4, TIGIT, and TIM-3 [116, 117].

### Speculative functions of m^6^A modification in TIE

In addition to the mechanisms discussed above, many other signaling pathways can modulate TIE, including metabolic alterations, acquisition of stemness, and epithelial-mesenchymal transition (EMT). m^6^A modification has been proven to play crucial roles in these pathways. Therefore, we speculate that m^6^A may also impact TIE via these pathways, which are discussed in the following section.

#### Aerobic glycolysis and TIE

Despite aerobic conditions, tumor cells prefer to produce energy through glycolysis rather than aerobic oxidation, along with more lactic acid generation, known as the Warburg effect [118]. Excess lactic acid acidifies the TME, endows tumor cells with stronger viability and aggressiveness, and has been identified as an important therapeutic target [119]. More importantly, the acidic TME impairs the immune response by weakening cytotoxic T cell function, blocking DC maturation, and enhancing helper cell activities [120]. In melanoma, *ALKBH5* knockdown effectively promotes sensitivity to anti-PD-1 treatment. Mechanistically, during anti-PD-1 treatment, ALKBH5 promotes lactate generation and consequent accumulation of Tregs and MDSCs in TME by stabilizing *Mct4/Slc16a3* mRNA, which is a pivotal enzyme mediating the transmembrane transport of lactate [121]. Many studies have found that m^6^A modifications play critical roles in regulating glycolysis.

**Fig. 4:**
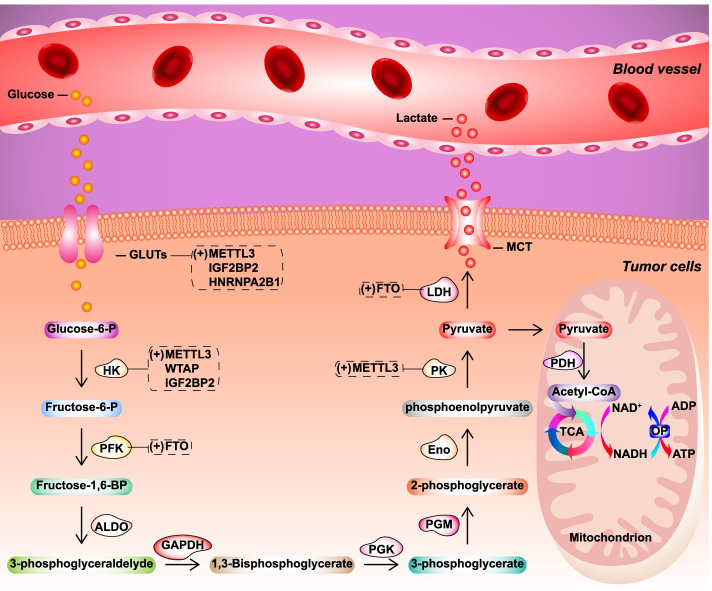
Overview of the regulation of tumor immune escape-associated glycolytic enzymes by *N*^6^-methyladenosine modification in tumor cells. The afferent blood delivers glucose to tissues, where it reaches the cells by diffusion. Glucose is taken up by specific glucose transporters (GLUTs), which are first converted to glucose-6-phosphate by hexokinase (HK) and then to pyruvate by various enzymes, including glucose phosphate isomerase (GPI), phosphofructokinase (PFK), aldolase (ALDO), glyceraldehyde-3-phosphate dehydrogenase (GAPDH), phosphoglycerate kinase (PGK), phosphoglycerate mutase (PGM), enolase (Eno), pyruvate kinase (PK), pyruvate dehydrogenase (PDH), and lactate dehydrogenase (LDH). ALDO, aldolase; Eno, enolase; GAPDH, glyceraldehyde-3-phosphate dehydrogenase; GLUT, glucose transporter; GPI, glucose phosphate isomerase; HK, hexokinase; LDH, lactate dehydrogenase; PDH, pyruvate dehydrogenase; PFK, phosphofructokinase; PGK, phosphoglycerate kinase; PGM, phosphoglycerate mutase; PK, pyruvate kinase

The glycolysis process in tumor cells and the regulation of glycolysis-related enzymes by m^6^A are summarized in Fig. 4 and Table 2. Glucose, the feedstock for glycolysis, is transported into the cells by glucose transporters (GLUTs), which are membrane proteins on the cell surface. Abnormal expression of GLUTs promotes glucose intake and glycolysis. In colorectal cancer (CRC), METTL3 promotes glycolysis and cancer progression in an m^6^A-dependent manner. METTL3-mediated m^6^A modification promotes *SLC2A1* (GLUT1) and *hexokinase 2* expression through IGF2BP2/3-dependent mRNA stability regulation, and activates glycolysis [122]. METTL3 was also shown to promote CRC progression by activating the m^6^A/GLUT1/mTORC1 pathway. Mechanistically, METTL3 promoted *GLUT1* translation in an m^6^A-dependent manner, increasing glucose uptake and lactate production and further activating the mTORC1 signaling pathway [123].

**Table 2:** The functions and mechanisms of m^6^A modification on pathways affecting TIE

Regulator	Cancer	Mechanism	Functional classification	Refs.
METTL3	CRC	Stabilizes *HK2* and *GLUT1*.	Enhances glycolysis to promote CRC progression.	[122]
METTL3	CRC	Promotes *GLUT1* translation.	Promotes CRC tumorigenesis by activating m^6^A/GLUT1/mTORC1 axis.	[123]
IGF2BP2	PC	Stabilizes *GLUT1.*	Promotes glycolysis and proliferation of PC.	[124]
HNRNPA2B1	PC	Promotes *GLUT1* expression.	Promotes glycolysis and proliferation of PC cell.	[125]
METTL3	CC	Promotes *HK2* stability.	Enhances CC aerobic glycolysis and tumorigenesis.	[126]
WTAP	lymphoma	Promotes *HK2* stability.	Enhances lymphoma cell proliferation.	[127]
WTAP	GC	Stabilizes *HK2.*	Promotes GC cell proliferation and glycolytic capacity.	[128]
IGF2BP2	glioblastoma	Promotes *HK2* mRNA stability.	Enhances glioblastoma aerobic glycolysis.	[129]
FTO	leukemia	Promotes stability of *PFKP* and *LDHB* mRNA.	Enhances aerobic glycolysis of leukemia cells.	[130]
METTL3	CC	Promotes the translation elongation and mRNA stability of *PDK4.*	Promotes CC cell glycolysis and proliferation.	[131]
METTL3	HCC	METTL3/IGF2BP1-mediated m^6^A stabilizes *LNCAROD.*	Promotes HCC cell glycolysis, proliferation, migration, invasion and chemoresistance via METTL3/IGF2BP1/LNCAROD/PKM2 pathway.	[132]
METTL3	HCC	Promotes *HIF-1α* expression.	Promotes the metabolic reprogramming and malignant biological behaviors of HCC cells.	[133]
YTHDC2	CRC	Promotes *HIF-1α* translation.	Promotes CRC metastasis.	[134]
METTL3	CRC	Stabilizes *SOX2*.	Promotes cell self-renewal, stem cell frequency and migration in vitro and suppresses CRC tumorigenesis and metastasis in vivo.	[135]
METTL3	glioblastoma	Promotes *SOX2* stability.	Promotes the GSC maintenance and glioma cell differentiation.	[136]
METTL3/14, FTO	glioblastoma	Promotes *AMAD19* expression.	FTO promotes and METTL3 inhibits GSCs growth and self-renewal.	[137]
METTL3	glioblastoma	Stabilizes *SRSF3/6/11*.	Promotes the growth and self-renewal of GSCs.	[138]
METTL3	glioblastoma	Stabilizes *NOTCH1* and *HES1*.	Promotes GSC maintenance and glioma progression.	[139]
METTL3	bladder cancer	Promotes *AFF4* expression.	Promotes self-renewal of bladder cancer stem cells.	[140, 141]
METTL3	cutaneous squamous cell carcinoma	Promotes *ΔNp63* expression.	Promotes cutaneous squamous cell carcinoma cell stem-like properties.	[142]
METTL3	CRC	Stabilizes *CBX8* mRNA.	Promotes stemness and suppresses chemosensitivity of CRC.	[143]
METTL3	LC	Promotes *RMRP* stability.	Promotes the CSCs properties and EMT, which promote the resistance to radiation therapy and cisplatin.	[144]
METTL3	kidney cancer	Promotes *ABCD1* translation.	Promotes cell migration, spheroid formation and tumor growth.	[145]
METTL3	oral squamous cell carcinoma	Downregulates *p38* expression.	Promotes stem-like capacities in oral squamous cell carcinoma cells.	[146]
METTL14	leukemia	Enhance *MYB* and *MYC* mRNA stability and translation.	Promotes self-renewal of LSCs.	[147]
METTL14	EC	Upregulates *miR-99a-5p* by promoting pri-mir-99a processing.	Promotes CSCs persistence and the radio-resistance.	[148]
FTO	leukemia	Promotes *MYC* and *LILRB4* expression.	Promotes leukemia CSC maintenance and immune evasion.	[39]
ALKBH5	glioblastoma	Demethylates *FOXM1* to enhance its expression.	Promotes proliferation of GSCs.	[149]
ALKBH5	BC	Stabilizes *NANOG.*	Promotes BC stem cells enrichment.	[150, 151]
YTHDF1	OC	Promotes translation of m^6^A-modified *TRIM29*.	Promotes the stem cell-like phenotype of cisplatin-resistant OC cells.	[152]
YTHDF2	glioblastoma	Stabilizes *MYC* and *VEGFA*.	Promotes GSCs growth.	[153]
YTHDF2	leukemia	Destabilizes *TNFRSF2.*	Promotes LSC development and AML initiation.	[154]
YTHDF2	liver cancer	Promotes *OCT4* translation.	Promotes liver cancer stem cell phenotype and metastasis.	[155]
IGF2BP1	BC	Stabilizes *c-My*c mRNA.	Promotes BC stem cells self-renewal and tumorigenesis.	[156]
IGF2BP1	liver cancer	Stabilizes *MGAT5.*	Promotes the liver cancer stem cell phenotype.	[157]
IGF2BP2	PC	Stabilizes *DANCR* mRNA.	Promotes cell proliferation and stemness-like properties.	[158]
METTL3	GC	Stabilizes *ZMYM1.*	Promotes EMT and metastasis of GC.	[159]
METTL3	BC	Upregulating *MALAT1* expression.	Promotes EMT, migration, and invasion of BC.	[160]
METTL3	EC	Promotes the splicing of precursor *miR-20a-5p* to produce mature miRNAs.	Promotes EMT, invasion, and metastasis of EC.	[161]
METTL3	CRC	Stabilizes *HMGA1*.	Induces EMT and promotes proliferation, migration, and invasion in vitro and tumor growth and metastasis in vivo.	[162]
METTL3	CRC	Promotes *circ1662* expression.	Promotes EMT and metastasis of CRC.	[163]
METTL3	LC	Increases the splicing of precursor *miR-143-3p* to facilitate its biogenesis.	Promotes EMT and brain metastasis of LC.	[164]
METTL3	LC	Promotes *YAP1* mRNA stability.	Promotes LC cell proliferation, metastasis, and EMT.	[165]
METTL3	LC	Destabilizes *ZBTB4*.	Promotes EMT and malignancy of LC.	[166]
METTL3	HCC	Determines the fate of the *HSP5* transcript to process it into circHSP5.	Promotes EMT and CSC phenotypes.	[167]
METTL3	HCC	Stabilizes *Snail* mRNA.	Promotes HCC metastasis.	[168]
METTL3	PC	Promotes *ITGB1* mRNA stability.	Promotes bone metastasis of PC.	[169]
FTO	GC	Promotes *ITGB1* expression.	Promotes GC metastasis.	[170]
ALKBH5	uveal melanoma	Promotes *FOXM1* expression.	Promotes uveal melanoma metastasis by inducing EMT.	[171]
IGF2BP2	renal cancer	Stabilizes *DUXAP9.*	Promotes renal cancer cells proliferation and motility capacities in vitro and induces EMT.	[172]

*Abbreviations: AML* acute myeloid leukemia, *BC* breast cancer, *CC* cervical cancer, *CRC* colorectal cancer, *CSC* cancer stem cell, *EC* esophageal cancer, *GC* gastric cancer, *GLUT* glucose transporter, *GSC* glioblastoma stem-like cell, *HCC* hepatocellular carcinoma, *HK* hexokinase, *LC* lung cancer, *LDHB* lactate dehydrogenase B, *LSC* leukemia stem cell, *OC* ovarian cancer, *PC* pancreas cancer, *PDK4* pyruvate dehydrogenase kinase 4, *PKM2* pyruvate kinase isoform M2, *PFKP* phosphofructokinase platelet

Glycolysis is triggered after glucose is taken into cells. Its progression is maintained by several enzymes, such as hexokinase (HK), aldose enzyme, glucose phosphate isomerase (GPI), phosphofructokinase (PFK), aldolase (ALDO), glyceraldehyde-3-phosphate dehydrogenase (GAPDH), phosphoglycerate kinase (PGK), phosphoglycerate mutase (PGM), enolase (Eno), pyruvate kinase (PK), pyruvate dehydrogenase (PDH), and lactate dehydrogenase (LDH), which have been reported to be regulated by m^6^A modification (Fig. 4, Table 2). In cervical cancer (CC), METTL3 promotes proliferation and aerobic glycolysis of CC cells. METTL3 binds to the 3′-UTR of *HK2* mRNA to catalyze m^6^A modification, which is recognized by YTHDF1 to enhance *HK2* stability [126]. In general, m^6^A is shown to enhance glycolysis by regulating the expression of key enzymes. METTL3 promotes glucose uptake, lactate generation, and ATP level, which can be reversed by METTL3 knockdown. Downregulation of METTL3 suppresses tumor growth and glycolysis progression in tumor cells and xenograft mouse models. Since m^6^A has the function of reprogramming energy metabolism, we hypothesized that this process is accompanied by reprogramming TME. Consequently, targeting m^6^A may effectively overcome TIE and effectively treat a variety of cancers in combination with immunotherapy.

In tumor cells, hypoxia-inducible factor-1α (HIF-1α) has been reported to enhance glycolysis by upregulating glycolysis-related enzymes, inhibiting PDH and mitochondrial oxidative phosphorylation, and subsequently leading to TME acidification and TIE [173–175]. METTL3 promotes glycolysis and malignant biological behaviors of HCC cells by methylating *HIF-1α* [133]. Moreover, the m^6^A reader YTHDC2 can promote metastasis by enhancing *HIF-1α* translation in CRC [134]. However, further studies are required to determine whether this occurs by regulating m^6^A modification. Although m^6^A modification has been shown to affect glycolysis from multiple pathways, its effect on TIE deserves further exploration.

#### Cancer stem cell-like characteristics and TIE

Cancer stem cells (CSCs) are subpopulations with strong self-renewal ability, pluripotency, and tumorigenicity that are closely related to tumor initiation, metastasis, drug resistance, and recurrence. They can also obtain TIE through their immunomodulatory properties [176]. Aberrant m^6^A modifications are related to the initiation and maintenance of CSC-like phenotypes in tumor cells (Table 2). FTO promotes the self-renewal of leukemia stem cells (LSCs) and TIE, which can be reversed by small-molecule FTO inhibitors (FTOis) [39]. FTOis (CS1 and CS2) impairs LSC self-renewal properties by occupying the catalytic pocket of FTO to inhibit its demethylase activity, which leads to increased m^6^A abundance and decreased expression of *MYC* and *CEBPA*. In contrast, FTO promotes *LILRB4* expression, an immune checkpoint gene, by demethylating *LILRB4* to inhibit the m^6^A-mediated degradation. FTOis effectively overcomes TIE and sensitizes acute myeloid leukemia (AML) cells to T cell cytotoxicity. More importantly, LSCs share numerous characteristics with hematopoietic stem cells (HSCs). Therefore, eliminating LSCs without damaging HSCs as much as possible is the key to treating leukemia. Knockdown of *YTHDF2* promotes HSC self-renewal to enhance HSC ex vivo expansion without any noticeable lineage bias or leukemic potential by stabilizing *Tal1* mRNA [177]. This may help overcome the limitations of umbilical cord blood in treating leukemia, as there are insufficient HSCs in a single human umbilical cord blood unit. METTL3 promotes glioblastoma stem cell (GSC) self-renewal and tumorigenesis by enhancing m^6^A modifications to stabilize transcripts of some CSC-related genes, such as *AMAD19*, *SOX2*, *SRSF3/6/11*, *NOTCH1*, and *HES1* [136–139]. Furthermore, YTHDF2 promotes glioblastoma stemness by stabilizing *MYC* and *VEGFA* in an m^6^A-dependent manner [153]. In a word, m^6^A promotes the initiation and maintenance of CSC-like phenotypes in tumor cells. Inhibitors targeting m^6^A can effectively reverse stem-like phenotype and block TIE. Further studies are needed to dissect whether m^6^A reverses TIE by affecting CSCs. What is more, none of the existing immunotherapy approaches selectively targets CSCs. Therefore, understanding this mechanism will provide a solution for optimizing or identifying new immunotherapy strategies and their combinations.

#### Epithelial-mesenchymal transition and TIE

EMT is a cellular reprogramming process that detaches epithelial cells from each other and the underlying basement membrane, eventually transforming them into mesenchymal cells. EMT increases the developmental and metastatic potential of cancer cells and drug resistance [178]. An increasing number of studies have shown that EMT can regulate antitumor immunity. For example, in melanoma cells, SNAIL-induced EMT stimulates the secretion of TGF-β and thrombospondin 1, which promotes the formation of Treg cells and impairs the antigen-presenting capacity of DCs [179]. This attenuates the immunogenicity of melanoma cells and their sensitivity to immunotherapy, which can be restored by inhibiting SNAIL [179]. Furthermore, EMT promotes immunosuppression and TIE in BC cells. Tumors derived from more mesenchymal carcinoma cell lines express lower MHC-I and higher PD-L1 and contain within their stroma Treg cells, M2 macrophages, and exhausted CD8^+^ T cells than tumors derived from more epithelial carcinoma cell lines [180]. The above evidence indicates that EMT can promote immunosuppression of tumor cells and obtain TIE.

EMT promotes the expression of the EMT-inducing transcription factors (TFs) ZEB, SNAIL, and TWIST, which activate mesenchymal state-related genes (*N-cadherin*, *vimentin*, *fibronectin*, *β1* and *β3 integrins*, and *matrix metalloproteinases*) and resilient epithelial state-related genes (*E-cadherin*, *epithelial cell adhesion molecule*, *occludins*, *claudins*, *α6β4 integrins*, and *cytokeratins*). Studies have demonstrated that upregulated m^6^A levels promote EMT in cancer cells, such as HeLa cells (CC), HepG2 cells (liver cancer), Huh7 cells (liver cancer), and A549 cells (LC). Increased m^6^A modification promotes *SNAIL* mRNA translation, which can be enhanced or inhibited by *ALKBH5* or *METTL3* knockdown. Depletion of METTL3 blocks invasion, migration, and EMT of cancer cells and tumor metastasis [181]. Furthermore, METTL3-mediated m^6^A also potentiates EMT by regulating integrin-β1 and ZMYM1/E-cadherin pathway [159, 169]. In addition to these EMT-inducing TFs, m^6^A also induces EMT by regulating the expression of other genes, such as *MALAT1*, *HMGA1*, *circ1662*, *miR-20a-5p*, *FOXM1*, *DUXAP9*, *YAP*, and *ZBTB4* [160–163, 165, 166, 171, 172]. In addition to its roles in regulating RNA stability and translation processes, m^6^A has been found to promote EMT by promoting the splicing of precursor miRNAs. For example, METTL3-mediated m^6^A modification promotes the splicing of precursor miR-143-3p to produce mature miRNAs, thus contributing to enhanced EMT via the METTL3/miR-143-3p/VASH1 pathway in LC cells [164]. In liver cancer, METTL3 determines the fate of the HSP5 transcript to process it into circHSP5 rather than mRNA. Increased circHSP5 acts as a miR-370 sponge to promote HMGA2 expression and potentiate EMT [167]. Abnormal m^6^A promotes EMT by regulating EMT-inducing TFs and other genes; however, its role in TIE remains unclear. Whether regulation of EMT through m^6^A modification can attenuate TIE and promote immunotherapy efficacy remains to be further explored.

### Targeting m^6^A modification in cancer therapy

The m^6^A levels of specific RNA transcripts have been shown to influence tumor development [182]. Therefore, inhibitors targeting m^6^A regulators may be effective new approaches for tumor therapy (Table 3). Rhein derived from the rhizome of *Rheum palmatum*, which was identified as the first competitive inhibitor target Alkb subfamily by structure-based in silico high-throughput screening and further structural optimization [183]. It competitively bounds to FTO or AlkB catalytic domain to form a complex and prevent the recognition of m^6^A substrates inside cells, which can increase the cellular m^6^A on mRNA. Rhein shows several bioactivities; however, its cellular targets remain largely unknown [196, 197]. Rhein also inhibits ALKBH2 activity, which is responsible for demethylating *N*^1^-methyladenosine modification in vitro. Furthermore, rhein inhibits other Fe^2+^- and 2OG-dependent hydroxylases by high-throughput screening in the NIH Molecular Libraries Probe Development Center Network (MLPCN) program, including the Jumonji domain containing 2A and 2E (JMJD2) histone demethylases and prolyl-4-hydroxylase. Rhein binding to FTO still has the possibility of multiple orientations. The crystal structure of small molecules in complexes with FTO has not been determined. In addition, increased m^6^A distributions due to rhein in cells may result from the direct inhibition of cellular demethylation via FTO or other members of nucleic acid demethylases. Characterizing the cell specificity of rhein will also be important to demonstrate its use as a cellular probe for nucleic acid demethylase in future studies. Rhein has been shown to have anticancer activity against various cancers. For example, rhein inhibited tumor growth in 4 T1 BC xenografts [184]. Another inhibitor, meclofenamic acid (MA), was identified by a high-throughput fluorescence polarization assay and selectively inhibited FTO demethylation of m^6^A over ALKBH5 [186]. This slightly higher selectivity for FTO in the Alkb subfamily mainly depends on its structure. MA is neither a mimic of 2OG nor a chelator of iron, and the structural complex of MA bound to FTO is a β-hairpin motif which is a part of the nucleotide recognition lid (NRL) for providing hydrophobic interactions with MA. In contrast to FTO, ALKBH5 lacks this part of NRL loop, resulting in leakage upon MA binding. In addition, MA could not inhibit ALKBH2 and ALKBH3 which have this region of NRL. The existence of the hydrophilic and bulky residues in the part of the NRL might significantly disturb the inhibitor MA binding to ALKBH2 and ALKBH3. Based on this structural complex, it should be possible to design more optimized analogs for FTO specificity and potency. In addition, MA increases m^6^A abundance in HeLa cells in an FTO activity-dependent manner. In glioblastoma, MA2 (an ethyl ester form of MA) significantly inhibits GSC growth and self-renewal, effectively suppresses GSC-induced tumorigenesis, and prolongs the lifespan of GSC-grafted mice [137]. In later studies, MA2 was shown to enhance the effect of the chemotherapeutic drug temozolomide on the suppression of glioma cell proliferation [187]. Subsequently, many FTOis can effectively inhibit the proliferation, promote the apoptosis of AML cells in vitro and suppress tumor growth in vivo, including FB23-2, R-2HG, CS1, and CS2 [39, 191, 192].

**Table 3:** Inhibitors of m^6^A regulators in cancer treatment

Drugs	Regulator	Cancer	Function	Structure	Refs.
Rhein	FTO	BC	Suppresses tumor growth of BC in vivo.		[183, 184]
			Augments antiproliferative effects of atezolizumab based on BC regression.		[185]
MA2	FTO	glioblastoma	Inhibits GSCs growth and self-renewal in vitro, and tumor growth in vivo.		[137, 186]
			Enhances the effect of the chemotherapy drug temozolomide on suppressing proliferation of glioma cells.		[187]
MO-I-500	FTO	BC	Inhibits the survival and/or colony formation of a triple-negative inflammatory BC cell line.		[188–190]
FB23-2	FTO	AML	Suppresses proliferation and promotes the differentiation/apoptosis of AML cells and in vitro, and inhibits tumor growth in vivo.		[191]
R-2HG	FTO	AML	Inhibits cell growth, promotes cell cycle arrest and apoptosis of leukemia cells.		[192]
CS1	FTO	AML, BC, PC, and glioblastoma	Suppresses CSC maintenance and immune evasion of AML.		[39]
CS2					
BTYNB	IGF2BP1	LC, OC	Inhibits proliferation and anchorage-independent growth of IGF2BP1-positive cancer cells. Blocks tumor cells’ growth and spread in xenograft tumors. Synergizes with palbociclib at low concentrations of both compounds.		[193, 194]
STM2457	METTL3	AML	Inhibits AML cells growth and promotes differentiation and apoptosis in vitro, and disrupts engraftment and prolonged survival in vivo.		[195]

*Abbreviations: AML* acute myeloid leukemia, *BC* breast cancer, *CSC* cancer stem cell, *GSC* glioblastoma stem cell, *LC* lung cancer, *MA* meclofenamic acid, *OC* ovarian cancer, *PC* pancreatic cancer, *R-2HG* R-2-hydroxyglutarate

In addition to FTO, other m^6^A regulators are key targets for treating m^6^A-associated tumors. BTYNB is screened as a potent and selective inhibitor among 16,000 small molecules via the fluorescence anisotropy-based assay, which inhibits IGF2BP1 binding to a specific high-affinity binding site in the coding region stability determinant of *c-Myc* mRNA. BTYNB effectively reduces the expression of c-Myc mRNA and protein. It inhibits proliferation and anchorage-independent growth of IGF2BP1-positive cancer cells [193]. Furthermore, IGF2BP1 acts as the dependent E2F-transcription super-enhancer. The E2F pathway is regulated by IGF2BP1 in an m^6^A-dependent manner. BTYNB blocks E2F1 expression at both mRNA and protein levels, as well as inhibits E2F/IGF2BP1-driven gene expression by reducing the binding of IGF2BP1 to E2F1 mRNA. BTYNB effectively blocks tumor cells’ growth and spread in xenograft tumors. Moreover, BTYNB synergizes with palbociclib at low concentrations of both compounds, suggesting that BTYNB is beneficial for combination therapy to impinge tumor cell proliferation [194]. However, BTYNB showed the ability to reduce IGF2BP1-dependent stabilization of mRNAs, and its putative off-target effects are unknown. Therefore, we hypothesized that BTYNB might interfere with the interaction between IGF2BP1 and m^6^A sites. STM2457 is a highly potent and selective inhibitor of METTL3 without disrupting the METTL3–METTL14 complex, identified by high-throughput screening. STM2457 effectively inhibits AML cell growth and promotes differentiation and apoptosis while reducing m^6^A levels of known AML-related mRNAs to block their translation and expression. In in vivo studies, STM2457 disrupted engraftment and prolonged survival in AML mouse models [195]. In addition to these competitive inhibitors, there are other ways to inhibit the functions of m^6^A regulators. First, the catalytic capacity of METTL3 depends on the heterodimer structure formed with METTL14; hence, it would be reasonable to design inhibitors based on protein-protein interaction strategies. Second, structural analysis of METTL3 suggested that the binding sites of the substrate SAM are merged into a large pocket; therefore, the development of bi-substrate inhibitors occupying both binding sites may be another effective strategy. Third, the proteolysis-targeting chimera strategy is a promising technology to degrade target proteins via proteasomes. Targeted m^6^A modification for clinical application is still in the initial stage and has not yet entered clinical trials. However, with increasing knowledge of the function and mechanism of m^6^A modification in cancer, it is expected that drugs targeting m^6^A modification will be developed and applied to clinical treatment in the near future.

### Targeting m^6^A modification in TIE

Regulation of the antitumor immune response by m^6^A RNA methylation is still in its infancy. However, recent studies have shown the possibility of combining immunotherapy with newly developed m^6^A regulator inhibitors for cancer therapy. Rhein significantly enhances the antiproliferative effects of atezolizumab (an anti-PD-L1 antibody) in 4 T1 BC xenografts. Moreover, the proportion of CD8^+^ T cells in the spleen and tumor is significantly increased in the combination therapy group and is significantly different from that in the monotherapy groups. Serum levels of TNF-α and IL-6 are significantly elevated in the rhein and combination therapy groups. In addition, the levels of various apoptotic factors in the tumor tissues are significantly higher in the combination treatment group [185]. Two FTOis are identified by structure-based virtual screening, CS1 and CS2, which show strong antitumor activity in leukemia. Inhibition of FTO by CS1 or CS2 significantly suppresses LSC self-renewal and promotes the immune response by suppressing the expression of the immune checkpoint gene *LILRB4*. They reverse TIE by abolishing the FTO-induced stability of *LILRB4* mRNA and enhancing the sensitivity of AML cells to T-cell cytotoxicity [39]. ALKBH5 promotes lactate generation and Tregs and MDSC accumulation by stabilizing *Mct4/Slc16a3* mRNA in melanoma cells. A specific ALKBH5 inhibitor, ALK-04, is identified by in silico screening of compounds using the X-ray crystal structure of ALKBH5 and by performing structure-activity relationship studies on a library of synthesized compounds. ALK-04 potently enhances anti-PD-1 therapy response in in vivo experiments [121]. These studies show not only the inhibition of m^6^A demethylases as a potential anticancer target but also their potential to reverse TIE. Although inhibitors of m^6^A modification regulators have been demonstrated to have anticancer roles by modulating tumor immunity, none have been tested in a clinical setting.

## Conclusions and perspectives

In this review, we outline the different TIE mechanisms and summarize the increasing excitement surrounding the development of the regulatory roles of m^6^A modification involved in these mechanisms. Due to many new related discoveries in recent years, updating the academic progress of m^6^A modification is still necessary. Here, we summarize the research progress of m^6^A modification and the core function of m^6^A in TIE. Although m^6^A modification is directly related to TIE, the exact molecular mechanism underlying its regulation remains unclear. The complex TIE mechanism in tumor cells is the cause of the low response rate to immunotherapy. Therefore, m^6^A modification may be considered a potential candidate target for targeting the TIE mechanism and is expected to be vital to overcoming immunotherapy-related challenges.

In the pathogenesis of tumors, m^6^A modification regulates RNA splicing, decay, nuclear export, stability, and translation, promotes the expression of oncogenes, or inhibits the expression of tumor suppressor genes, thereby inducing TIE. The putatively dynamic and reversible characteristics of m^6^A modifications make them attractive in the field of anticancer therapy. In light of new findings on the physiological roles of m^6^A regulators. At present, it is more important to clarify the ‘real’ regulator and their specific physiological function in certain cancers. This will help to explore the clear mechanism of m^6^A in TIE and target it more accurately. m^6^A RNA demethylase inhibitors have shown the potential to enhance immunotherapy [39, 121, 185]. The m^6^A methyltransferases METTL3 and METTL14 also show antitumor functions by reprogramming macrophages [198, 199]. SAM, the methyl donor for RNA, has shown anticancer activity in various cancer types by targeting histone methylation and DNA hypomethylation. It is unknown whether SAM inhibits tumor growth and metastasis by upregulating m^6^A RNA methylation levels or promoting immunotherapy. This means that m^6^A agonists, not just inhibitors, may also inhibit the growth of certain tumors. In addition, m^6^A modification exhibits cellular heterogeneity; that is, the same writer, eraser, and reader proteins may have different biological functions in different cells. This may cause it to act in the opposite manner in tumor or immune-related cells. Furthermore, other hypotheses could explain the paradox. First, m^6^A regulators may function independently of their m^6^A catalytic activity. Second, as recognized by different readers, genes modified by m^6^A modification can undergo different fates. Third, the location of m^6^A modifications in different regions of the same mRNA transcript may lead to different results. Therefore, additional studies are required to clarify the roles of m^6^A modifications in TIE, including the contributions of specific regulators, targets, modes of action, and TME.

Further studies are required to determine whether m^6^A modification is likely to provide new insights into identifying patients with tumors susceptible to specific drugs, identifying prognostic indicators, and developing targeted drugs. We believe that this approach is highly personalized. Utilizing a detailed understanding of m^6^A levels and each patient’s immune status will be the basis for the next step in immunotherapy. In addition, m^6^A status, the TME-infiltration characteristics, and the immune system can change over time, especially during treatment. Therefore, continuous monitoring of m^6^A regulators and immune markers is essential for developing and adjusting treatment regimens.

Both DNA and histone methylation inhibitors have been shown to enhance the efficacy of immunotherapy. Therefore, the mechanism by which m^6^A modification interacts with DNA and histone epigenetics to regulate gene expression and whether there is a potential association between m^6^A modification and other types of methylation remains unclear. In addition, based on the theories and mechanisms obtained from these studies, we explored whether m^6^A inhibitors combined with other epigenetic drugs can synergistically promote immunotherapy. Moreover, dual inhibitors targeting both m^6^A regulators and immune checkpoints may achieve better efficacy and fewer side effects.

With the continuous development of this field, it will be necessary to continue to study the mechanism of m^6^A modifications leading to TIE as a theoretical basis and simultaneously accelerate clinical trials to translate the theories obtained. In this way, the outcomes of patients with cancer can be further improved.

## Data Availability

Not applicable.

## Abbreviations

3′ UTR: 3′ untranslated region
5′ UTR: 5′ untranslated region
ALKBH5: Alkb homolog 5
ALDO: Aldolase
AML: Acute myeloid leukemia
APC: Antigen-presenting cell
APM: Antigen processing and presenting machinery
BC: Breast cancer
CC: Cervical cancer
CLIP: Cross-linking and immunoprecipitation
COX2: Cyclooxygenase 2
CRC: Colorectal cancer
CTL: Cytotoxic T lymphocyte
CTLA4: Cytotoxic T lymphocyte antigen 4
CSC: Cancer stem cell
DC: Dendritic cell
DFS: Disease-free survival
EC: Esophageal cancer
EMT: Epithelial-mesenchymal transition
Eno: Enolase
FTO: Fat mass and obesity-associated protein
FTOi: FTO inhibitor
GAPDH: Glyceraldehyde-3-phosphate dehydrogenase
GLUT: Glucose transporter
GPI: Glucose phosphate isomerase
GSC: Glioblastoma stem cell
HCC: Hepatocellular carcinoma
HIF-1α: Hypoxia-inducible factor-1α
HK: Hexokinase
HLA-I: Class I human leukocyte antigens
HSC: Hematopoietic stem cell
ICI: Immune checkpoint inhibition
icSHAPE: In vivo click selective 2-hydroxyl acylation and profiling experiment
IDO: Indoleamine 2,3-dioxygenase
IGF2BP: Insulin-like growth factor 2 mRNA binding protein
IL-10: Interleukin-10
JMJD2: Jumonji domain containing 2
KH: K-homology
LC: Lung cancer
LDH: Lactate dehydrogenase
LSC: Leukemia stem cell
m^6^A: *N* ^6^-methyladenosine
m^6^ACE-seq: m^6^A-Crosslinking-Exonuclease-sequencing
m^6^Am: *N* ^6^,2′-O-dimethyladenosine
MA: Meclofenamic acid
MDSC: Myeloid-derived suppressor cell
MeRIP-seq: Methylated RNA immunoprecipitation sequencing
mESC: Mouse embryonic stem cell
METTL3: Methyltransferase-like protein 3
MHC: Major histocompatibility complex
miCLIP: m^6^A individual-nucleotide-resolution cross-linking and immunoprecipitation
MLPCN: Molecular Libraries Probe Development Center Network
MTC: Methyltransferase complex
NRL: Nucleotide recognition lid
OS: Overall survival
PC: Pancreatic cancer
PCIF1: Phosphorylated CTD interacting factor 1
PDH: Pyruvate dehydrogenase
PD-L1: Programmed death ligand 1
PD-1: Programmed cell death protein 1
PGE_2_: Prostaglandin E_2_
PFK: Phosphofructokinase
PGK: Phosphoglycerate kinase
PGM: Phosphoglycerate mutase
PK: Pyruvate kinase
RBP: RNA binding protein
RRM: RNA-recognition motif
SAM: S-adenosyl methionine
tAg: tumor antigen
TAM: Tumor-associated macrophages
TCR: T-cell receptor
TF: Transcription factor
Th1: Type 1 helper T cell
TIE: Tumor immune escape
TIGIT: T cell immunoreceptor with immunoglobulin and ITIM domain
TIM3: T-cell immunoglobulin and mucin-domain containing 3
TME: Tumor microenvironment
TNF: Tumor necrosis factor
Treg: CD4^+^ regulatory T cell
tRNA: Transfer RNA
WTAP: Wilms tumor 1-associated protein
ZCCHC4: Zinc finger CCHC-type containing 4
